# Melatonin and doxorubicin synergistically enhance apoptosis via autophagy-dependent reduction of AMPKα1 transcription in human breast cancer cells

**DOI:** 10.1038/s12276-021-00675-y

**Published:** 2021-09-28

**Authors:** Quynh Hoa Tran, Dang Hieu Hoang, Minhyeok Song, Wonchae Choe, Insug Kang, Sung Soo Kim, Joohun Ha

**Affiliations:** 1 Department of Biochemistry and Molecular Biology, Graduate School, College of Medicine, Kyung Hee University, Seoul, 130-701 Republic of Korea; 2 Department of Biotechnology, Ho Chi Minh city University of Food Industry, Ho Chi Minh, Vietnam

**Keywords:** Biochemistry, Diseases

## Abstract

Doxorubicin is one of the most effective agents used to treat various cancers, including breast cancer, but its usage is limited by the risk of adverse effects, including cardiotoxicity. Melatonin, a natural hormone that functions as a major regulator of circadian rhythms, has been considered a supplemental component for doxorubicin due to its potential to improve its effectiveness. However, the mechanisms and biological targets of the combination of melatonin and doxorubicin with respect to cancer cell death are not well understood. In the present study, we found that melatonin synergized with doxorubicin to induce apoptosis of breast cancer cells by decreasing the expression of AMP-activated protein kinase α1 (AMPK α1), which acts as a critical survival factor for cancer cells. This cotreatment-induced reduction in AMPKα1 expression occurred at the transcriptional level via an autophagy-dependent mechanism. The synergistic effects of the combined treatment were evident in many other cancer cell lines, and melatonin was also highly effective in inducing cancer death when combined with other cancer drugs, including cisplatin, 5-fluorouracil, irinotecan, and sorafenib. AMPKα1 expression was decreased in all of these cases, suggesting that reducing AMPKα1 can be considered an effective method to increase the sensitivity of cancer cells to doxorubicin treatment.

## Breast cancer: Melatonin could boost efficacy of chemotherapy

Combining the chemotherapy drug doxorubicin with the natural hormone melatonin reduces levels of a key enzyme and boosts treatment efficacy. Doxorubicin is a valuable treatment option for breast cancer but can have toxic side effects. Recent research suggests that taking melatonin in combination with chemotherapy drugs can increase treatment efficacy, but the underlying mechanisms are unclear. Joohun Ha at Kyung Hee University in Seoul, South Korea, and co-workers examined the effects of combining doxorubicin with melatonin on multiple breast cancer cell lines. They found that the synergistic activity of doxorubicin and melatonin reduced AMP-activated protein kinase (AMPK α1) levels in breast cancer cells. AMPKα1 is involved in cellular energy metabolism, and is known to promote cancer cell survival. By reducing AMPKα1 levels, the melatonin increased cancer cell sensitivity to doxorubicin, leading to cancer cell death.

## Introduction

Breast cancer is one of the three most common cancers worldwide, along with lung and colon cancer^1,2^. It is the most common malignancy and the leading cause of cancer-related death among females worldwide^1^. Breast cancer has become a global public health problem due to its complex etiology and poor response to treatment^3^. Doxorubicin, which is one of the most effective anthracycline agents used to treat breast cancer, can be used at all cancer stages as a curative agent for early and metastatic breast cancer^2^, even in patients with a high risk of relapse^4^. For many years, doxorubicin has been used as a first-line treatment for not only most patients with breast cancer^2^ but also those with many types of soft tissue sarcoma^5,6^. Although doxorubicin is an effective chemotherapeutic agent against multiple types of malignancies, its application has been limited by the risk for cardiotoxicity^7^.

Melatonin (*N*-acetyl-5-methoxytryptamine) is an indolic compound that is synthesized and secreted mainly by the pineal gland and plays a central role in regulating circadian and seasonal biorhythms in humans^8^. Melatonin has shown chemotherapeutic potential in many forms of human cancer^9–15^ and can increase the efficacy of anticancer drugs (e.g., doxorubicin, cisplatin, epirubicin, and bleomycin) by regulating many different signaling pathways^16–22^. Therefore, melatonin has been considered a potential supplementary component in chemotherapy to reduce the therapeutic doses and, thus, the adverse effects of anticancer drugs. However, the mechanisms by which melatonin synergizes with anticancer drugs are still unclear.

AMP-activated protein kinase (AMPK) is a heterotrimeric protein kinase composed of a catalytic subunit (α) and two regulatory subunits (β and γ) and plays a central role in the regulation of energy homeostasis^23^. The activity of AMPK is highly regulated at the protein level in response to changes in cellular energy status via allosteric regulation of adenine nucleotides (e.g., AMP, ADP, and ATP) and/or phosphorylation by upstream kinases, including LKB1 and calcium/calmodulin-dependent protein kinase kinase β^24^. There are multiple isoforms of each subunit: the α1 catalytic subunit is ubiquitously expressed, whereas α2 shows a highly tissue-specific expression pattern that includes the heart, skeletal muscle, and liver^25^. The role of AMPK has been intensively investigated under various metabolic conditions, but its function and activity in the presence of anticancer drugs or melatonin remain largely unresolved. There is no consensus on the role of AMPK in regulating cell viability under doxorubicin treatment^26–28^, and our previous study suggested that the isoforms of AMPKα play opposing roles in determining cell fate under doxorubicin treatment^29^.

In the present study, we examined the potential of melatonin to enhance the efficacy of doxorubicin in breast cancer cell lines. We demonstrate that the combination of melatonin and doxorubicin markedly induces apoptosis of breast cancer cells by reducing AMPKα1 messenger RNA (mRNA) at the transcriptional level in an autophagy-dependent manner. We also examined the effects of this combined treatment in various cancer cell lines and the effects of melatonin and several other anticancer drugs.

## Materials and methods

### Reagents and antibodies

Dulbecco’s modified Eagle’s medium (DMEM) and RPMI-1640 were purchased from Gibco (Grand Island, NY, USA). The breast cancer (MCF-7, MDA-MB157, MDA-MB231), colorectal carcinoma (HTC116), lung adenocarcinoma (A549), gastric adenocarcinoma (AGS), hepatocellular carcinoma (HepG2, Huh7), and mouse embryonic fibroblast (MEF) cell lines used in this study were purchased from American Tissue Culture Collection (ATCC, Manassas, VA, USA). *Ampkα*1^−/−^ knockout MEFs were a generous gift from Dr. B. Viollet (INSERM, Paris, France). Fetal bovine serum (FBS) was purchased from Corning (Corning, NY, USA). Antibodies against phospho-acetyl-CoA carboxylase α-Ser^79^, cleaved caspase-3, caspase-7, cleaved caspase-7, poly (ADP-ribose) polymerase (PARP), p62, and microtubule-associated protein 1A/1B-light chain 3B (LC-3B) were purchased from Cell Signaling Technology (Danvers, MA, USA). Antibodies against AMPKα1 and AMPKα2 were obtained from R&D Systems (Minneapolis, MN, USA). Antibodies against human influenza hemagglutinin and PARP were obtained from Santa Cruz Biotechnology (Santa Cruz, CA, USA). Anti-β-actin was purchased from Proteintech (Rosemont, IL, USA). Horseradish peroxidase (HRP)-conjugated rabbit anti-mouse and goat anti-rabbit secondary antibodies were purchased from Thermo Fisher (Waltham, MA, USA). G-418 (Geneticin) was purchased from Duchefa Biochemie (Haarlem, Netherlands). Melatonin (5-methoxy-*N*-acetyltryptamine), doxorubicin, irinotecan, cisplatin, and 5-fluorouracil (5-FU) were purchased from Sigma-Aldrich (St. Louis, MO, USA). Sorafenib and 3-methyladenine (3-MA) were purchased from Santa Cruz Biotechnology (Dallas, TX, USA). Small interfering RNAs (siRNAs) targeting AMPKα1 and ATG5 and nontargeting siRNAs (si-ctl) were purchased from GenePharma (Zhangjiang Hi-Tech Park, Shanghai, China). The siRNA transfection reagent INTERFERin^®^ was purchased from Polyplus-transfection^®^ (New York, NY, USA). The DNA transfection reagent *Trans*IT-X2® was obtained from Mirus Bio LLC (Madison, WI, USA).

### Cell cultures

MDA-MB-157, MDA-MB-231, HepG2, and MEFs were maintained in DMEM. Huh7, MCF-7, HCT116, A549, and AGS were cultured in RPMI-1640. All media were supplemented with 10% FBS and antibiotics, and cultures were incubated at 37 °C in humidified air containing 5% CO_2_.

### Plasmid construction and transfection

The promoter region of the human AMPKα1 gene (~1.7 kb) was amplified by PCR and inserted into the pGL3-basic reporter vector. MDA-MB-157 and MCF-7 cells were cultured in a growth medium and transfected with plasmids using *Trans*IT-X2® transfection reagent according to the manufacturer’s instructions (Mirus, Madison, WI, USA). All plasmid constructs were confirmed by sequencing.

### Establishment of stable overexpression cell lines

MDA-MB-157 and MCF-7 cells were transfected with pcDNA3-Ha*-*AMPK*α*1 or pcDNA3 vector (negative control). After 24 h, successfully transfected clones were selected with G-418 (200 μg/ml) for 2 weeks.

### RNA isolation and RT-PCR

RNA samples were extracted using TRIzol (Life Technologies, Grand Island, NY, USA). Reverse transcription-polymerase chain reaction (RT-PCR) was performed with 1 μg of extracted RNA by using a complementary DNA (cDNA) Synthesis Kit (Doctor Protein, Seoul, Korea). PCR was performed using *Taq* polymerase, primers against AMPKα1 or glyceraldehyde 3-phosphate dehydrogenase (GAPDH), and 1 μg of cDNA. The human AMPKα1 and GAPDH primer sequences were as follows: AMPKα1, forward (5′-CATGAAGAGGGCCACAATCA-3′) and reverse (5′-GGGCTTGTCGCCAAATAGAA-3′); and GAPDH, forward (5′-TGGGCTACACTGAGCACCAG-3′) and reverse (5′-ACCACCCTGTTGCTGTAGCC-3′). The primers for the PCR analysis of mouse AMPKα1 and GAPDH were as follows: AMPKα1, forward (5′-GATCGGCCACTACATCCTGG-3′) and reverse (5′-GATGTGAGGGTGCCTGAACA-3′); and GAPDH, forward (5′-GGAAGGGCTCATGACCACAGTCC-3′) and reverse (5′-CGACGGACACATTGGGGGTAGGA-3′). The level of each mRNA was normalized to that of GAPDH.

### Reporter gene assay

Cells were seeded in 12-well culture plates at 5 × 10^4^ cells/well and incubated for 24 h. Plasmids were transfected into cells with *Trans*IT-X2® according to the manufacturer’s instructions. After 24 h of transfection, cells were treated with the indicated drugs for 24 h. Then, luciferase activity was determined by mixing 20 μl of cell extract with 80 μl of luciferase assay reagent (Promega, Madison, WI, USA), and relative light units were measured using a Synergy HTX luminescence plate reader (BioTek, Winooski, VT, USA).

### Cell death assay

Apoptotic cells were detected with a MUSE Annexin V & Dead Cell Kit and a Muse cell analyzer (both from Merck Millipore, Danvers, MA, USA) according to the manufacturer’s instructions. The apoptotic ratio was determined by identifying four populations: (i) cells not undergoing detectable apoptosis, which were Annexin V (−) and 7-amino actinomycin D (AAD) (−); (ii) early apoptotic cells, which were Annexin V (+) and 7-AAD (−); (iii) late apoptotic cells, which were Annexin V (+) and 7-AAD (+); and (iv) cells that had died through a nonapoptotic pathway, which were Annexin V (−) and 7-AAD (+).

### Western blot analysis

Cells were washed with phosphate-buffered saline and lysed in lysis buffer (150 mM NaCl, Tris-HCl, 50 mM, pH 7.4, 1.0% Triton X-100, 0.1% sodium dodecyl sulfate (SDS), 5 mM EDTA, 10 mM NaF, and 2.0 mM Na_3_VO_4_) containing a protease inhibitor cocktail tablet (sc-29130; Santa Cruz Biotechnology). The lysates were cleared via centrifugation (21,000 × *g* at 4 °C for 15 min), and the supernatant from each sample was collected. The protein concentration was determined using Bradford’s solution at a ratio of 1:100 (cell lysate:Bradford solution). Equal amounts of protein (10 μg) were subjected to SDS-polyacrylamide gel electrophoresis through 8–13% gels. Proteins were transferred to a polyvinylidene fluoride membrane, and the membrane was washed with TBST solution (150 mM NaCl; 10 mM Tris-HCl, pH 7.6; and 0.1% Tween-20). The membrane was blocked with 5% bovine serum albumin in TBST solution for 1 h, washed with TBST, and incubated with the appropriate primary antibody (1:1000) overnight at 4 °C. The membrane was then washed and incubated with HRP-conjugated secondary antibody (1:10,000) for 1 h at room temperature. The Western blot bands were visualized using an enhanced chemiluminescence solution (Bio-Rad, Hercules, CA, USA). The signal intensity of primary antibody binding was quantified and normalized to that of the loading control (β-actin).

### Statistical analysis

The results are expressed as the means ± SEM. Each experiment was repeated at least three times with three samples, and the data were analyzed by two-tailed Student’s *t* test. *P* < 0.05 was considered significant. *P* values are indicated in the figures as follows: ***p < 0.005, **p < 0.01, and *p < 0.05; n.s. indicates not significant.

## Results

### Melatonin synergizes with doxorubicin to promote apoptosis and decrease AMPKα1 expression in breast cancer cells

**Fig. 1:**
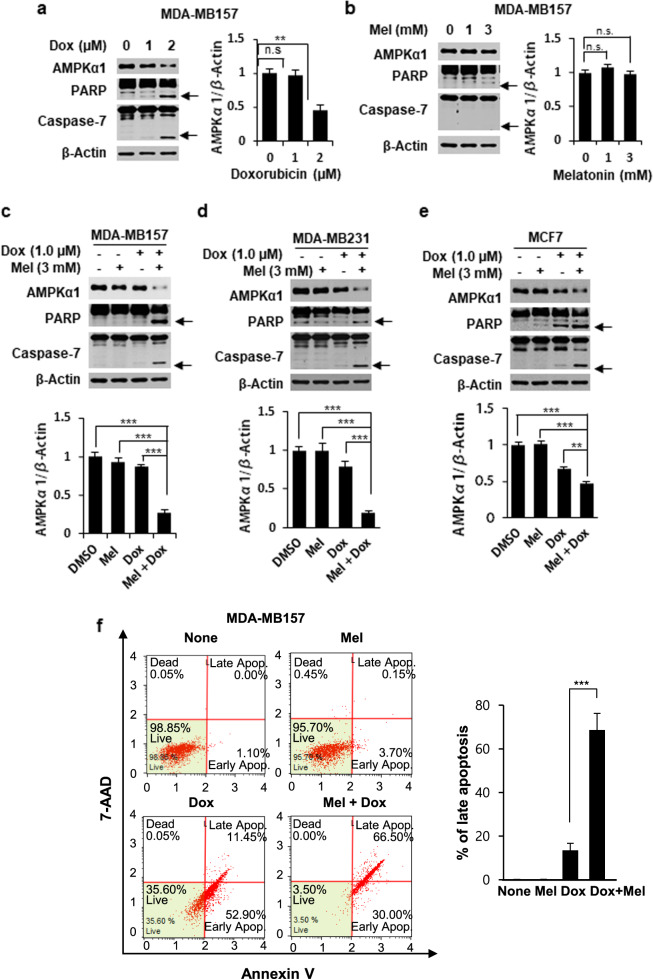
The effects of melatonin and doxorubicin on apoptosis and the level of AMPKα1 in breast cancer cells. MDA-MB-157 breast cancer cells were treated with the indicated concentrations of doxorubicin (**a**) or melatonin (**b**) for 24 h. MDA-MB-157 (**c**), MDA-MB-231 (**d**) and MCF-7 (**e**) cells were pretreated with 3 mM melatonin for 2 h, and then 1 µM doxorubicin was added for 24 h. The expression levels of AMPKα1, caspase-7, and PARP were examined via Western blot analyses, and the band intensity of AMPKα1 was quantified. The arrow indicates the cleaved form (**a–e**). The populations of 7-ADD and Annexin V double-positive MDA-MB-157 cells were measured by Muse cell analyzer (**f**). Dox doxorubicin, Mel melatonin. ***p < 0.005; **p < 0.01; *p < 0.05; n.s. not significant.

Initially, we examined the effects of doxorubicin and melatonin either alone or in combination on the protein levels of AMPKα1 and apoptotic markers (cleaved caspase-7 and cleaved PARP) in breast cancer cells. Doxorubicin treatment of MDA-MB-157 breast cancer cells increased the cleavage of caspase-7 and PARP and decreased the level of AMPKα1 in a dose-dependent manner (Fig. 1a). By contrast, melatonin showed no significant effect on these parameters at doses up to 3 mM (Fig. 1b). We then tested the combination of 1 μM doxorubicin and 3 mM melatonin, which were dosages that had no significant effect when applied alone. This combined treatment resulted in a significant induction of apoptosis and reduction in AMPKα1 expression in several different breast cancer cell lines, including MDA-MB-157, MDA-MB-231, and MCF-7 cells (Fig. 1c–e), whereas the same dose of doxorubicin with 1 mM melatonin did not show a synergistic effect. The synergistic effect of this combined treatment on apoptosis was further demonstrated via fluorescence-based analysis of 7-ADD and Annexin V double-positive cells (Fig. 1f). These results suggest that the combined treatment with doxorubicin and melatonin is highly effective for inducing apoptosis in breast cancer cells. Our data also indicate that the level of AMPKα1 is inversely correlated with the degree of apoptosis in response to this treatment.

### Knockdown of AMPKα1 promotes doxorubicin-induced apoptosis of breast cancer cells

**Fig. 2:**
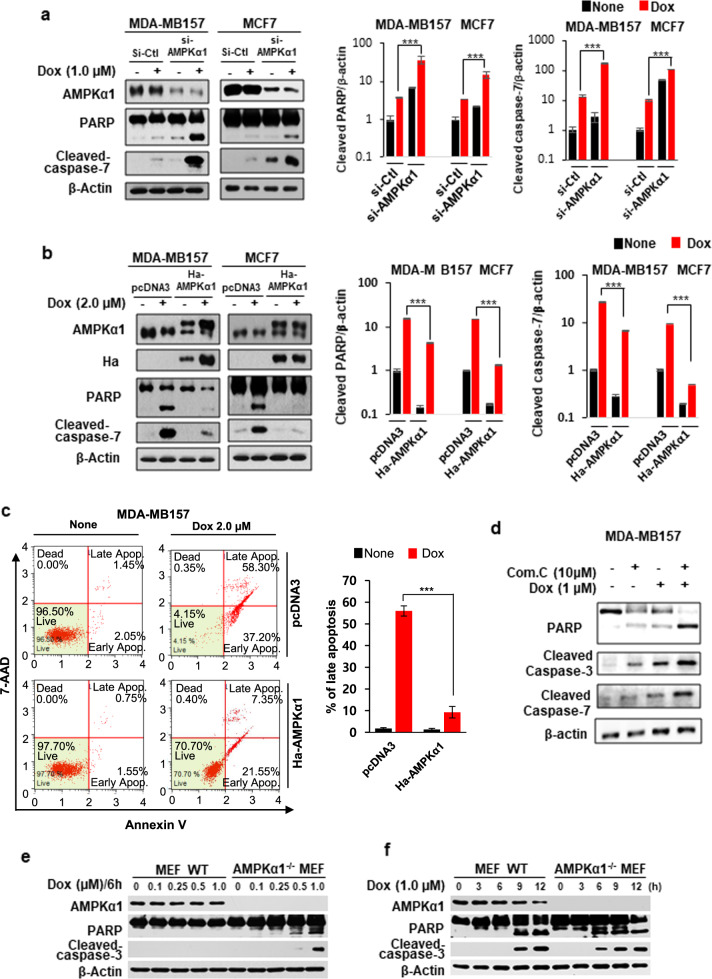
Knockdown of AMPKα1 promotes doxorubicin-induced apoptosis of breast cancer cells. MDA-MB-157 and/or MCF-7 breast cancer cells were transfected with siRNA targeting AMPKα1 (**a**) or an expression vector overexpressing HA-tagged AMPKα1 (**b**, **c**). The cells were then incubated with the indicated concentrations of doxorubicin for 24 h. Western blot analyses (**a**, **b**) or analyses of 7-ADD and Annexin V double-positive MDA-MB-157 cells (**c**) were performed. MDA-MB-157 cells were treated with doxorubicin and the AMPK inhibitor compound C for 24 h (**d**). Wild-type and *AMPKα*1^−/−^ MEFs were treated with the indicated concentrations of doxorubicin for 6 h (**e**) or with 1 µM doxorubicin for the indicated time periods (**f**). The expression levels of AMPKα1, PARP, and cleaved caspase-3 were then examined via Western blot analyses. si-Ctl nonspecific siRNA, si-AMPKα1 siRNA targeting AMPKα1, Com. C compound C. ***p < 0.005; **p < 0.01; *p < 0.05; n.s. not significant.

We next examined the role of AMPKα1 in regulating the fate of breast cancer cells under doxorubicin treatment, as this is poorly understood. Knockdown of AMPKα1 via siRNA in MDA-MB-157 and MCF-7 cells resulted in a marked augmentation of doxorubicin-induced apoptosis (Fig. 2a), whereas overexpression of AMPKα1 rendered these breast cancer cells highly resistant to doxorubicin-induced apoptosis (Fig. 2b, c). In accordance with the results of AMPKα1 knockdown, the combined treatment with doxorubicin and the AMPK inhibitor compound C synergistically induced the apoptosis of MDA-MB-157 cells (Fig. 2d). Moreover, AMPKα1-knockout MEFs (*Ampkα*1^−/−^) were more sensitive to doxorubicin than were wild-type MEFs (Fig. 2e, f). These results suggest that AMPKα1 plays a critical role in protecting breast cancer cells against doxorubicin-induced cytotoxicity, further indicating that AMPKα1 is a cellular factor that contributes to determining cellular susceptibility to doxorubicin.

### Melatonin synergistically enhances the chemotherapeutic effect of doxorubicin via induction of autophagy

**Fig. 3:**
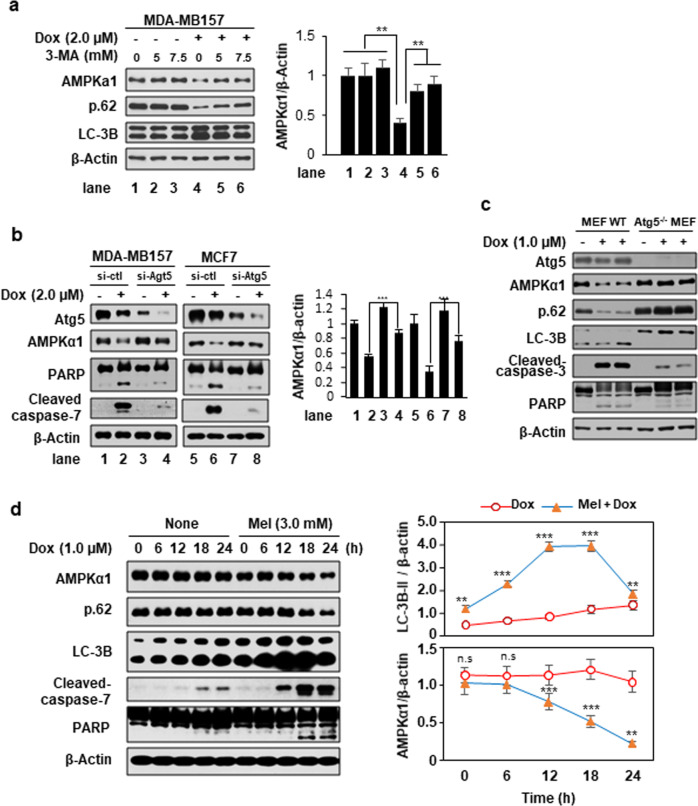
Melatonin and doxorubicin synergistically enhance autophagy in breast cancer cells. **a** MDA-MB-157 cells were pretreated with the indicated concentrations of 3-MA for 30 min and then cultured in the presence or absence of doxorubicin for 24 h. **b** MDA-MB-157 and MCF-7 cells were transfected with siRNA targeting Atg5 (si-Atg5) and then treated with doxorubicin for 24 h. **c** Wild-type and *Atg*5^−/−^ MEFs were treated with doxorubicin for 12 h. **d** MDA-MB-157 cells were treated with doxorubicin for the indicated durations in the presence or absence of melatonin (3 mM). Under these conditions, the expression levels of target proteins were examined via Western blot analyses. ***p < 0.005; **p < 0.01; *p < 0.05; n.s. not significant.

The role of autophagy in the regulation of doxorubicin sensitivity among cancer cells, along with the relevant underlying mechanisms, has become the subject of substantial interest^30,31^. Here, we observed that treatment of MDA-MB-157 cells with 2 μM doxorubicin, which induced apoptosis and reduced the level of AMPKα1, also induced autophagy, as indicated by an increase in the level of LC-3B and a decrease in the level of p62/SQSTM1 (p62) (Fig. 3a). Furthermore, the AMPKα1 level was recovered in these cells by adding the autophagy inhibitor 3-MA (Fig. 3a), suggesting that the downregulation of AMPKα1 is tightly associated with autophagy induced by doxorubicin treatment. To further study this apparent association, we blocked doxorubicin-induced autophagy via siRNA-mediated knockdown of autophagy-related gene 5 (*Atg*5) in MDA-MB-157 and MCF-7 cells and examined the level of AMPKα1 and the degree of apoptosis (Fig. 3b). *Atg*5 is well known to play a critical role in inducing autophagy. Our present results showed that doxorubicin-induced apoptosis was significantly blocked and that the level of AMPKα1 was not reduced when autophagy was blocked by Atg5 knockdown (Fig. 3b). Similar phenomena were observed in *Atg*5-knockout MEFs (*Atg*5^−/−^) (Fig. 3c). Moreover, the combination of 1 μM doxorubicin and 3 mM melatonin robustly induced autophagy in MDA-MD-157 breast cancer cells (Fig. 3d). Taken together, these results suggest that doxorubicin treatment induces autophagy to contribute to breast cancer cell apoptosis and that the expression of AMPKα1, which protects against doxorubicin-induced cytotoxicity, is reduced in an autophagy-dependent manner.

### Melatonin and doxorubicin synergistically inhibit AMPKα1 transcription

**Fig. 4:**
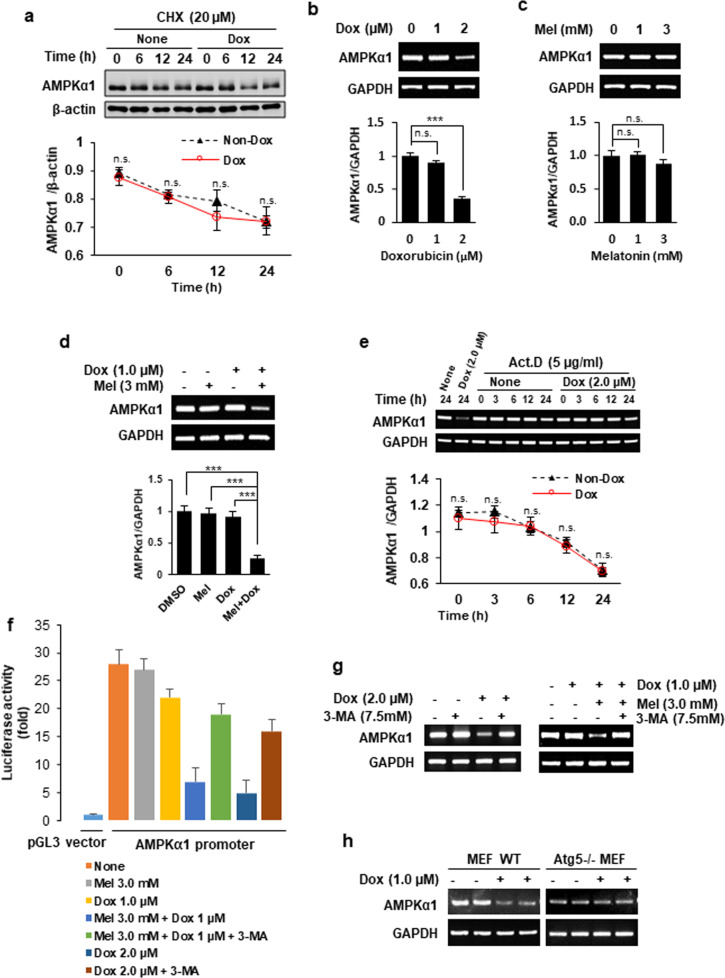
Melatonin and doxorubicin synergistically reduce AMPKα1 at the transcriptional level. **a** MDA-MB-157 cells were cultured in the presence or absence of 2.0 µM doxorubicin for the indicated time periods in the presence of cycloheximide. The protein levels of AMPKα1 were examined via Western blot analyses. **b**–**d** MDA-MB-157 cells were treated with the indicated concentrations of doxorubicin (**b**), melatonin (**c**), or a combination of 3 mM melatonin and 1 µM doxorubicin (**d**) for 24 h. The cells were subjected to quantitative analysis of AMPKα1 mRNA. GAPDH served as a control. **e** MDA-MB-157 cells were cultured with or without doxorubicin (2.0 µM) in the presence of actinomycin D (Act. D) for the indicated durations. The mRNA levels of AMPKα1 were analyzed. **f** MDA-MB-157 cells were transfected with a pGL3 luciferase reporter vector containing a human AMPKα1 promoter (~1.7 kb). The cells were treated as indicated for 24 h, and luciferase activity was measured. MDA-MB-157 cells (**g**) or wild-type and *Atg*5^−/−^ MEFs (**h**) were treated as indicated for 24 h, and the levels of AMPKα1 mRNA were measured. ***p < 0.005; **p < 0.01; *p < 0.05; n.s. not significant.

In response to various nutritional and stressful stimuli, AMPK enzyme activity is rapidly regulated via allosteric regulation by AMP, ADP, and ATP, as well as by phosphorylation by upstream kinases; meanwhile, the protein level of AMPKα1 remains relatively constant^23^. Given our finding that the level of AMPKα1 was dramatically reduced by doxorubicin, we next examined whether AMPKα1 protein stability was affected by doxorubicin treatment. MDA-MD-157 breast cancer cells were incubated with the protein translation inhibitor cycloheximide in the presence or absence of doxorubicin, and we monitored the AMPKα1 protein level for 24 h (Fig. 4a). We did not observe a significant difference in the half-life of AMPKα1 protein under these conditions (Fig. 4a). Notably, however, our subsequent RT-PCR analysis revealed that the AMPKα1 mRNA level was significantly reduced in response to treatment with 2 μM doxorubicin (Fig. 4b), but not with melatonin (Fig. 4c). Melatonin treatment also potentiated the effect of 1 μM doxorubicin (which had no significant effect when applied alone) on the AMPKα1 mRNA level (Fig. 4d). We further examined the half-life of AMPKα1 mRNA under doxorubicin treatment by incubating cells with the transcription inhibitor actinomycin D in the presence or absence of 2 μM doxorubicin. Our results showed that doxorubicin did not affect the half-life of AMPKα1 mRNA (Fig. 4e). To examine whether doxorubicin regulates the transcriptional activity of AMPKα1, we cloned a human AMPKα1 promoter fragment (~1.7 kb) into the pGL3 luciferase reporter vector and transfected this construct into MDA-MD-157 breast cancer cells. In accordance with our findings with regard to the mRNA levels of AMPKα1 (Fig. 4b–d), doxorubicin treatment inhibited AMPKα1 promoter activity in a dose-dependent manner (Fig. 4f). Although treatment with either 1.0 µM doxorubicin or 3.0 mM melatonin alone had relatively little effect, the combination treatment markedly inhibited AMPKα1 promoter activity (Fig. 4f). The addition of 3-MA (an autophagy inhibitor) effectively blocked the effect of either 2 µM doxorubicin or the combination treatment comprising 1.0 µM doxorubicin and 3.0 mM melatonin on the promoter activity (Fig. 4f) and mRNA level (Fig. 4g) of AMPKα1. Moreover, the inhibitory effect of doxorubicin on AMPKα1 mRNA levels was not observed in *Atg*5^−/−^ MEFs (Fig. 4h). Collectively, these results suggest that doxorubicin decreases AMPKα1 mRNA at the transcriptional level in an autophagy-dependent manner and that melatonin potentiates this effect.

### Melatonin and doxorubicin cotreatment synergistically reduces AMPKα1 in various cancer cells

**Fig. 5:**
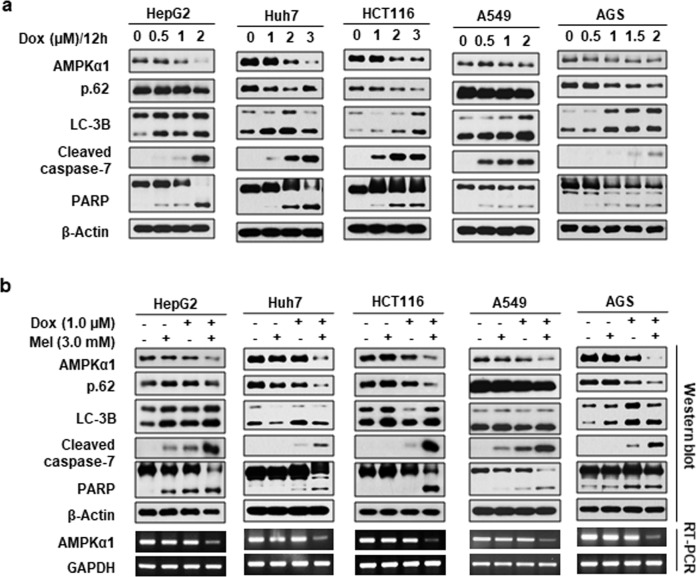
The synergistic effects of melatonin and doxorubicin on apoptosis, autophagy, and AMPKα1 mRNA expression in different cancer cells. Hepatocellular carcinoma (HepG2, Huh7), colorectal carcinoma (HCT116), lung adenocarcinoma (A549), and gastric adenocarcinoma (AGS) cell lines were treated with the indicated concentration of doxorubicin (**a**) or with a combination of melatonin and doxorubicin (**b**) for 24 h. The levels of the indicated proteins were examined via Western blot analysis (**a**, **b**), and the mRNA levels of AMPKα1 were examined by RT-PCR (**b**).

Doxorubicin chemotherapy is prominently used for not only breast cancer^2^ but also many other forms of cancer^5,6^. Accordingly, we next examined the effects of doxorubicin and melatonin on AMPKα1 levels in other cancer cell lines, including hepatocellular carcinoma (HepG2, Huh7), colorectal carcinoma (HCT116), lung adenocarcinoma (A549), and gastric adenocarcinoma (AGS) cell lines. Similar to our findings in breast cancer cells, doxorubicin treatment reduced AMPKα1 expression and induced autophagy and apoptosis in a dose-dependent manner (Fig. 5a), and combined treatment with 3.0 mM melatonin and 1.0 µM doxorubicin markedly reduced AMPKα1 mRNA levels and induced autophagy and apoptosis in all of the tested cancer cell lines (Fig. 5b).

### The combination of melatonin with several different anticancer drugs synergistically reduce AMPKα1

**Fig. 6:**
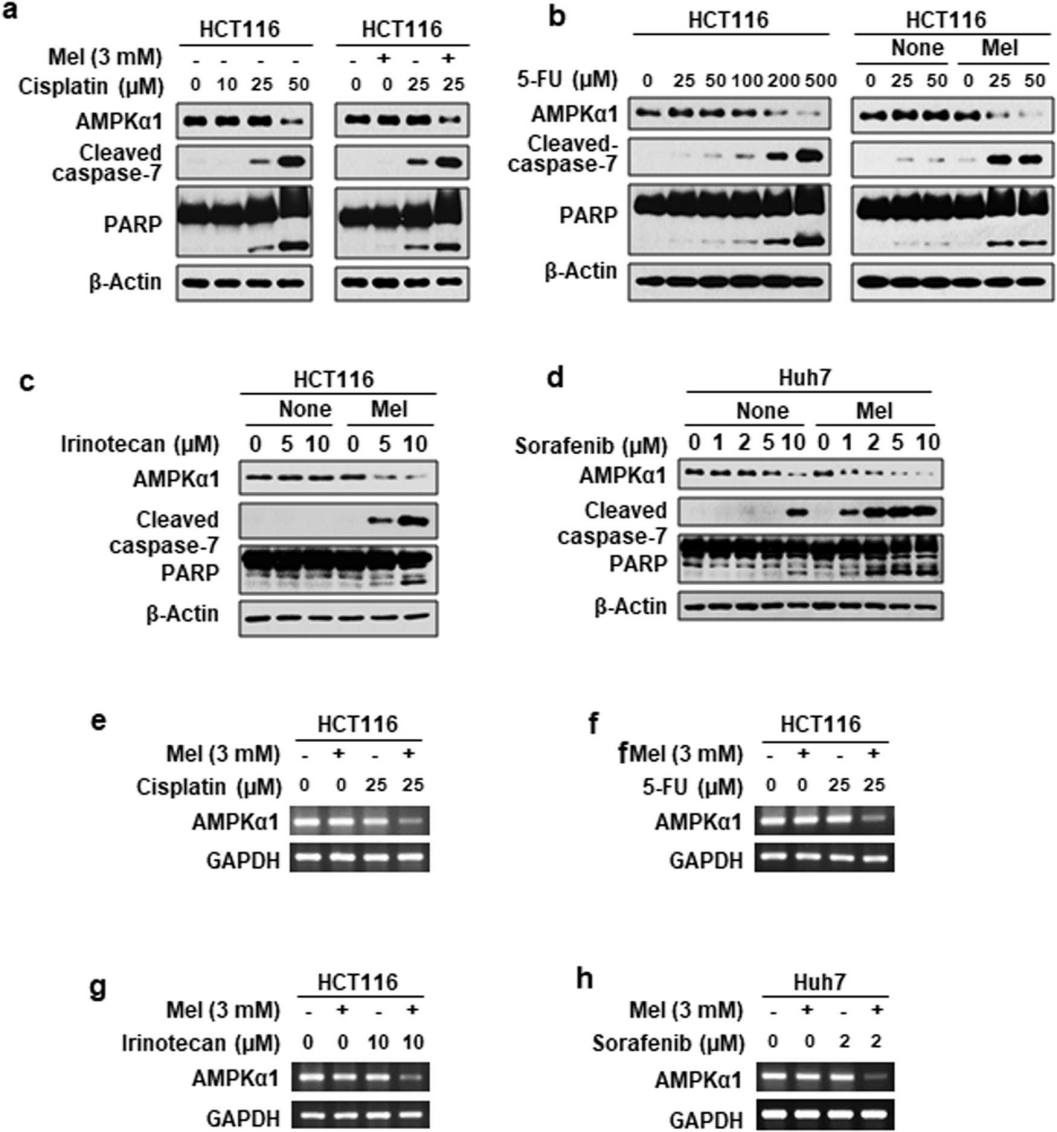
The synergistic effects of melatonin with other anticancer drugs. HCT116 cells were treated with the indicated concentrations of cisplatin (**a**, **e**), 5-FU (**b**, **f**), and irinotecan (**c**, **g**) in the absence or presence of melatonin for 24 h. Huh7 cells were treated with the indicated concentration of sorafenib in the absence or presence of melatonin for 24 h (**d**, **h**). Under these conditions, the levels of the indicated proteins (**a**–**d**) and mRNAs (**e**–**h**) were measured.

Finally, we examined the effects of combination treatment of melatonin with several other anticancer drugs, including cisplatin (Figs. 6a), 5-FU (Fig. 6b), irinotecan (Fig. 6c), and sorafenib (Fig. 6d), on AMPKα1 levels. Our results showed that melatonin potentiated the apoptosis-inducing effects of these anticancer drugs and that this effect was associated with significant reductions in the mRNA level of AMPKα1 (Fig. 6e–h).

## Discussion

The goal of chemotherapy is to promote cancer cell death without damaging noncancerous cells. Doxorubicin is an essential chemotherapeutic reagent for the treatment of breast cancer^2,4^ as well as of many other forms of cancer^5,6^, but its clinical application has been impeded by its risks for adverse effects, including cardiotoxicity^7^. Therefore, researchers are urgently seeking to improve the therapeutic efficacy of doxorubicin therapy while minimizing the adverse effects. Melatonin appears to be one of the most effective supplementary components that can satisfy this requirement, as it can reestablish the sensitivity of breast tumors to doxorubicin while protecting against doxorubicin-induced cardiotoxicity^32^. Combined treatment comprising doxorubicin and melatonin was previously demonstrated to have synergistic effects in inducing apoptosis in hepatoma^17^ and breast cancer^33^ cells. Melatonin also reportedly showed cardioprotective effects against several anticancer drugs^34^. Although multiple signaling pathways have been studied with the aim of understanding how melatonin synergizes with anticancer drugs, including doxorubicin^17–22^, the precise mechanisms remain highly unclear. In the present study, we observed that the combination of melatonin and doxorubicin reduced the level of AMPKα1, which acts as a critical survival factor for cancer cells; this phenomenon was seen in many different cancer cells, including breast cancer cells, and was also evident in cells subjected to combined treatment with melatonin and other cancer drugs. We, therefore, suggest that reducing AMPKα1 levels can be further developed as an adjuvant therapy to increase the sensitivity of cancer cells toward doxorubicin and thereby reduce its adverse effects. However, the reduction in the expression of a survival factor such as AMPKα1 in cardiomyocytes may provide unfavorable environments for cancer patients, and a combination therapy involving melatonin and doxorubicin requires careful analyses of their effects on cardiomyocytes as well as on cancer cells.

Although the role of AMPK in metabolic regulation is quite well established, its roles under genotoxic stress or during tumorigenesis appear to be quite contradictory. First, there is no consensus regarding whether AMPK is activated^26,27^ or inhibited by doxorubicin^28^. Second, there are conflicting reports regarding the role of AMPK in tumor metabolism. Some studies have demonstrated that AMPK promotes tumor cell survival by regulating NADPH homeostasis^35^ or by balancing glycolysis and mitochondrial metabolism^36^. However, AMPK was also reported to suppress tumor growth by negatively regulating the Warburg effect^37^. In the present study, we demonstrate that AMPKα1 acts as a survival factor for cancer cells in response to doxorubicin. In sharp contrast, AMPKα2 was reported to induce cell death under doxorubicin treatment in noncarcinoma cells^29^. AMPKα isoforms are therefore likely to exert opposing roles under genotoxic stress conditions. Given this, researchers should be careful to distinguish the roles of AMPKα isoforms when seeking to develop a novel chemotherapeutic strategy involving AMPK signaling pathways.

AMPK plays a central role as a cellular energy sensor in response to various conditions that alter the cellular energy level; the underlying mechanism involves rapid changes in its protein level via allosteric regulation^23^. Under ATP-depleting conditions, the accumulated AMP binds to the γ-subunit and activates AMPK. This interaction with AMP makes the kinase a better substrate for upstream kinases, leading to phosphorylation and the consequent full activation of AMPK. Intriguingly, we herein revealed that the level of AMPKα1 can be regulated at the transcriptional level in response to doxorubicin (Fig. 4). We previously reported that the transcription of AMPKα2 is robustly induced by E2F1 in noncarcinoma cells treated with doxorubicin^29^. Therefore, our present and previous results emphasize the complexity of AMPK regulatory mechanisms by revealing transcriptional regulation of AMPKα subunits. Additional work is needed to examine the mechanisms underlying the transcriptional regulation of AMPKα1.

Autophagy is generally considered to be a defense mechanism that maintains cell viability by recycling damaged cellular constituents under various conditions of cellular stress^38^. However, accumulating evidence suggests that excessive or dysregulated autophagy contributes to cell death^39^. It has also been reported that treatment with cytotoxic drugs, including doxorubicin, often results in the induction of autophagic cell death^31,40^, highlighting the complex signaling outcomes of autophagy. Accumulating data suggest that apoptosis-resistant cancer cells can undergo autophagy, and this induction of autophagic cell death has been suggested as a new strategy for cancer therapy^30^. Here, we observed that doxorubicin strongly induced autophagy that contributed to apoptosis; this apoptosis was significantly blocked in cells with defective autophagy (Fig. 3). Importantly, the mRNA levels and promoter activity of AMPKα1 were inhibited in an autophagy-dependent manner in our system (Fig. 4). To date, there is a lack of information on the specific transcription factor responsible for regulating the transcription of AMPKα1. Since the transcription of AMPKα1 was suppressed by doxorubicin in an autophagy-dependent manner, it would be highly intriguing to identify transcription factors that regulate AMPKα1 in an autophagy-dependent manner. For example, GATA4, which is a transcription factor known to be involved in cardiac development and function, is downregulated by doxorubicin^41^ and subjected to autophagy-dependent degradation^42^. We indeed examined this possibility, but GATA4 is unlikely to regulate the AMPKα1 promoter (Supplementary Fig. 1). Nevertheless, we cannot completely rule out the involvement of GATA4 because a relatively short region of the AMPKα1 promoter (~1.7 kb) was examined in this study. Further studies on the regulation of the AMPKα1 promoter would provide us with a highly intriguing insight into not only the regulatory mechanisms of AMPK but also potential applications for chemotherapy.

In conclusion, we herein suggest that AMPKα1 is a common survival factor for various cancer cells, that a reduction in AMPKα1 expression can be exploited as an adjuvant therapy to render many cancer cell types sensitive to anticancer drugs, and that this reduction further alleviates the adverse effects of anticancer drugs.

## Supplementary information

Supplementary Figure 1
