# Supplementary material for: Melatonin and doxorubicin synergistically enhance apoptosis via autophagy-dependent reduction of AMPKα1 transcription in human breast cancer cells
Source: Exp Mol Med. 2021 Sep 28;53(9):1413–22. doi: 10.1038/s12276-021-00675-y (PMC8492618; doi:10.1038/s12276-021-00675-y)
Supplement: Supplementary file 1 — Supplementary Figure 1 [file 12276_2021_675_MOESM1_ESM.pdf]

Supplementary Figure 1

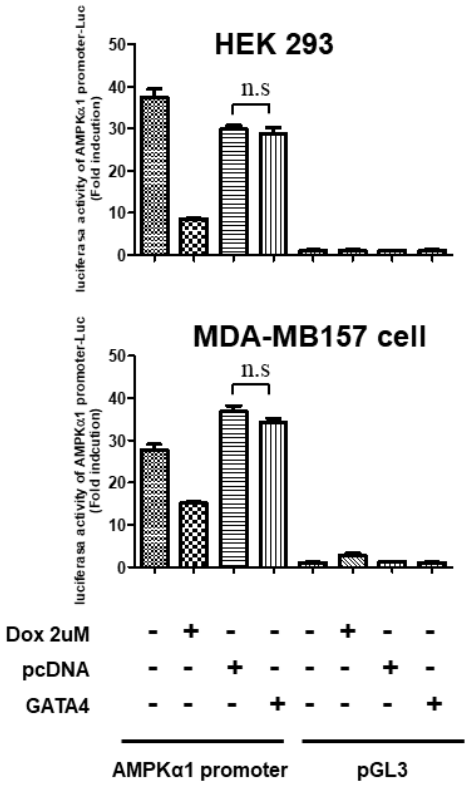

HEK293 cells or MDA-MB157 cells were co-transfected with a pGL3 luciferase reporter vector containing a human AMPKα1 promoter (~1.7 kb) and the pcDNA-GATA4 expression vector or treated with doxorubicin (2μM) for 24 h. Then, the luciferase activity was measured. n.s, not significant
